# Contribution of Intravital Neuroimaging to Study Animal Models of Multiple Sclerosis

**DOI:** 10.1007/s13311-022-01324-6

**Published:** 2023-01-18

**Authors:** Emeline Buttigieg, Anja Scheller, Bilal El Waly, Frank Kirchhoff, Franck Debarbieux

**Affiliations:** 1 Molecular Physiology, Center for Integrative Physiology and Molecular Medicine (CIPMM), University of Saarland, 66421 Homburg, Germany; 2 Institut des Neurosciences de la Timone (INT), Aix-Marseille Université, CNRS UMR7289, 13005 Marseille, France; 3 Centre Européen de Recherche en Imagerie Médicale (CERIMED), Aix-Marseille Université, Marseille, France; 4 Institut Universitaire de France (IUF), Paris, France

**Keywords:** Animal models, Multiple sclerosis, Intravital imaging, Multimodal microscopy, Neurodegenerative disease

## Abstract

Multiple sclerosis (MS) is a complex and long-lasting neurodegenerative disease of the central nervous system (CNS), characterized by the loss of myelin within the white matter and cortical fibers, axonopathy, and inflammatory responses leading to consequent sensory-motor and cognitive deficits of patients. While complete resolution of the disease is not yet a reality, partial tissue repair has been observed in patients which offers hope for therapeutic strategies. To address the molecular and cellular events of the pathomechanisms, a variety of animal models have been developed to investigate distinct aspects of MS disease. Recent advances of multiscale intravital imaging facilitated the direct in vivo analysis of MS in the animal models with perspective of clinical transfer to patients. This review gives an overview of MS animal models, focusing on the current imaging modalities at the microscopic and macroscopic levels and emphasizing the importance of multimodal approaches to improve our understanding of the disease and minimize the use of animals.

**Supplementary Information:**

The online version contains supplementary material available at 10.1007/s13311-022-01324-6.

## Introduction

With 2.5 millions of people affected with multiple sclerosis (MS) worldwide, MS is one of the most common neurodegenerative diseases [1]. There is an overall increase in incidence over recent years and a clear female preponderance. It is an inflammatory autoimmune disease of the CNS leading to loss of the myelin sheath and axonal degeneration causing progressive sensory-motor and cognitive disabilities in patients. The complete MS pathophysiology has not yet been elucidated, although potential environmental factors, including viral infections, and/or genetic variants have been associated with an increased risk to develop MS [2]. Infiltration of immune cells such as T cells into the CNS parenchyma elicits the inflammatory processes observed in relapsing–remitting phases, subsequently leading to degeneration and non-relapsing progression, i.e., secondary progressive MS [3]. Although conventional tissue staining techniques of animal and human specimen contributed to the pathophysiological understanding of the disease, the emergence of intravital microscopy (IVM) of animal models of MS has allowed significant advances. Two-photon (2P) non-linear scanning microscopy allowed to highlight cell dynamics and interactions at the lesion site in vivo. Moreover, the implementation of label-free imaging approaches such as Coherent Anti-Stoke Raman Scattering (CARS) or Spectral Confocal REflectance (SCoRE) microscopy has empowered the assessment of myelin status regarding MS evolution and provided insights into the mechanisms of myelin degeneration. Concurrently, preclinical research has enabled the development of new tracers to target cells of interest, known to be important to monitor disease evolution, with magnetic resonance imaging (MRI) or positron emission tomography (PET). Finally, to improve our current knowledge, new modalities will soon allow to further expand capabilities of imaging and analysis in the MS field. Novel three-photon (3P) microscopy provides access to image in deep tissue, while adaptative optics (AO) and artificial intelligence (AI) improve image resolution and analysis, hence the accessibility of fine cellular phenomena. Combining non-linear optical microscopy with clinical imaging modalities will simultaneously generate valuable information at micro- and macroscale levels in large animal models if not directly in patients.

### Animal Models of Multiple Sclerosis

Since more than 70 years, animal models have been essential to study the pathophysiology of MS. Even if they are not equivalent to the human disease, they are able to partially recapitulate the events associated with MS at a high level of reproducibility and thus helpful to address MS-related questions. Thereby, we obtained a detailed knowledge of cellular interactions and mechanisms underlying CNS demyelination or used them to test potential therapies. However, simultaneously, each model is restricted to distinct processes: the models of experimental autoimmune encephalomyelitis (EAE) exhibit neuroinflammation and immune system activation toward myelin, toxin-induced demyelination models are relevant to decipher de- and remyelination processes selectively targeting oligodendrocytes and virus-induced MS models start with axonal damage while induce inflammation leading to demyelination.

#### EAE Models

EAE models mimic clinical symptoms of MS patients as well as the time course of the disease. EAE is based on immunizing mice by a subcutaneous injection of a water-in-oil emulsion of myelin-associated peptides together with or without inactivated mycobacteria (complete or incomplete Freund’s adjuvant; CFA or IFA). The choice of the adjuvant has its relevance: IFA implies Th2 humoral response whereas CFA triggers Th1 cells mediated immunity with microglia activation releasing pro-inflammatory chemokines or reactive oxygen species (ROS) and subsequent activation of astrocytes [4, 5]. The addition of pertussis toxin is required to develop EAE by facilitating the access of immune cells into the CNS [6, 7]. According to mouse strains or the immunization peptides used, EAE models may differ in terms of symptom severity and time course of the disease. Mice can be immunized with myelin basic protein (MBP)- or proteolipid protein (PLP)-peptides, which in SJL/J mouse strain allow addressing the relapsing–remitting phase of MS [8, 9]. Immunization against the myelin-oligodendrocyte glycoprotein (MOG) instead induces a chronic and progressive form of EAE. Moreover, MOG-reactive T cells are more widely observed in patients than MBP- or PLP-reactive T cells, thereby making it the main EAE model to study the pathophysiology of MS [10, 11]. Demyelination is firstly triggered by the infiltration of autoreactive T cells into the CNS parenchyma, possibly activated by B cells [12, 13]. Together, these cells attack the myelin sheath in the early stages of the disease. When mice are not directly immunized against myelin peptides but rather inoculated with reactivated T cells or splenocytes from immunized mice, this is referred to as “passive EAE” or “adoptive-transfer EAE” models. This pathogenic transfer is enough to induce EAE-like symptoms to recipient naive mice [14, 15]. However, from an imaging point of view, this model suffers from the difficulty of localizing lesion sites and their time course during EAE progression.

#### Toxin-Induced Models

##### Lysolecithin

The ability of lysolecithin or lysophosphatidylcholine (LPC) to produce demyelination was first described in 1972 by Hall [16]. Due to its detergent activity, LPC solution at 1% concentration damages the myelin sheath in white matter tracts and selectively kills most of the mature myelinating oligodendrocytes (OLs) by dissolving their membrane [17]. Their higher sensitivity is possibly due to their inability to metabolize LPC compared to other cell types [18]. Usually, LPC is injected into the ventral horns of the spinal cord (SC), the corpus callosum (CC), or the optic nerve. However, delivery of LPC at the SC dorsal surface was proven to be valuable to differentiate demyelination induced axonal death/degeneration from the mechanical axonal degeneration induced by needle insertion during LPC injection [19]. The loss of OLs and demyelination occurs within 2–4 days after LPC injection and is followed by spontaneous remyelination 1 week later. Remyelination mainly relies on local oligodendrocyte precursor cells (OPCs) that proliferate and repopulate the lesion before differentiating into new myelinating OLs [20]. Although Schwann cells are cellular components of the peripheral nervous system may also contribute to remyelination to a certain extent, either by invading the lesioned parenchyma or by differentiating on site from OPCs [21, 22].

##### Ethidium Bromide

Involvement of Schwann cells strongly depends on the model used to induce the death of OLs. Ethidium bromide (EtBr) is a DNA intercalator that inhibits transcription and replication in the nucleus of all living cells causing their death [23]. Unlike LPC, EtBr is not selective to OLs. Its injection into the CNS causes the death of OLs but also that of other cells such as OPCs, astrocytes, and microglia. The lesion size is dose-dependent and the loss of axons is minimal [24]. Noteworthy, spontaneous remyelination is delayed compared to other animal models and demyelinated axons can still be observed six months after injection [25]. Although new OLs are generated, the remyelination largely involves Schwann cells. These lesions are indeed devoid of astrocytes, usually known to prevent Schwann cells recruitment.

##### Cuprizone

While LPC and EtBr must be applied topically or injected into the tissue to induce focal lesions, cuprizone (CPZ) is instead a systemically administered drug that produces reversible demyelination in relatively predictable brain regions such as CC, hippocampus, or cerebral and cerebellar cortex [26–28]. The addition of this copper chelator to mouse chow at 0.2–0.3% for a defined number of weeks alters selectively the homeostasis of OLs leading to their apoptotic death and subsequent loss of myelin sheath [29]. The susceptibility of OLs might be explained by mitochondrial disturbances causing their inability to support their high energetic demands for membrane synthesis [30, 31]. CPZ leads to early activation of microglia and strong astrogliosis preceding the demyelination [26]. The remyelination is achieved through migration and subsequent maturation of new OLs derived from OPCs and begins within a week after toxin arrest [32]. However, this model can be turned into a chronic-demyelinating model when CPZ treatment is extended to more than 12 weeks [33].

#### Viral-Induced Models

The growing evidence of a possible virus-triggered origin of MS brought up models of virus-induced demyelination to explore its potential etiology [34–36]. Usually, mice are infected with Theiler’s murine encephalomyelitis (TMEV) or murine hepatitis (MHV) viruses. The two major subgroups of TMEV strains are GDVII and BeAn strains and Daniel’s TMEV strains, the former causing fatal encephalomyelitis while the latter less virulent are mostly used [37]. Noteworthy, the TMEV model can only be induced in mice and the clinical outcomes depend on the mouse strains [38]. SLJ/V infected mice develop a progressive-like form of MS with the persistence of viral antigen presentation by microglia and OLs [39, 40]. Unlike in EAE where axonal degeneration is secondary to inflammation, in TMEV model, the axonal loss precedes demyelination despite the existence of important immune response triggered by the virus and involving many types of CD4^+^ and CD8^+^ cytotoxic T cells, B cells, and even macrophages [41, 42]. MHV infection is considered an MS-like models thanks to its ability to target OLs and to induce demyelination directly via OLs death [43, 44]. The viral-induced models initially used to study the efficiency of the CNS immune system to clear viruses also proved useful to study mechanisms of demyelination that are not primarily induced by autoreactive T and B cells [45].

### Intravital Non-Linear Optical Microscopy to Decipher the Pathophysiology of Multiple Sclerosis

**Fig. 1:**
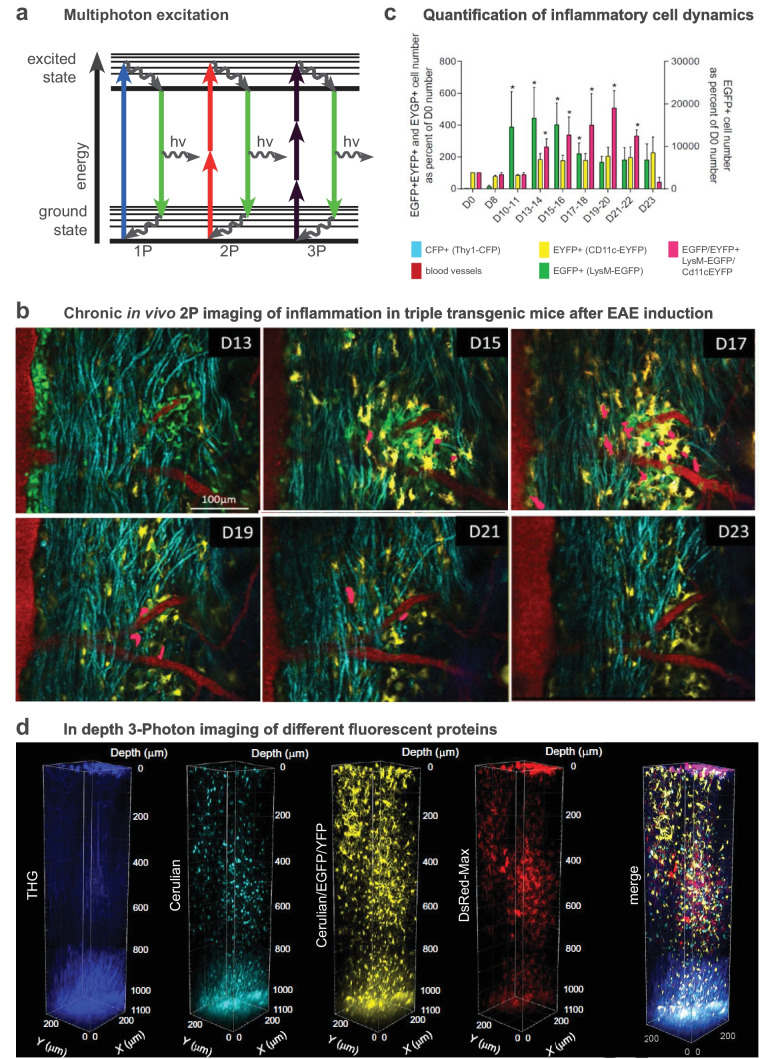
In vivo multi-photon microscopy to visualize inflammatory responses. **a** Principle of multi-photon microscopy. **b** In vivo visualization of immune cell dynamics in the spinal cord of a triple fluorescent mouse during EAE progression (**d**, day after EAE induction; modified from [57]). **c** Quantification of the same inflammatory plaque shown in **b**. **d** 3D-multicolor 3P- and third harmonic generation (THG) images of the cortex of a multifluorescent mouse illustrating the typical 1.2 mm imaging depth achieved at 1340 nm excitation as well as the THG signal collected from corpus callosum myelin (modified from [84])

**Fig. 2:**
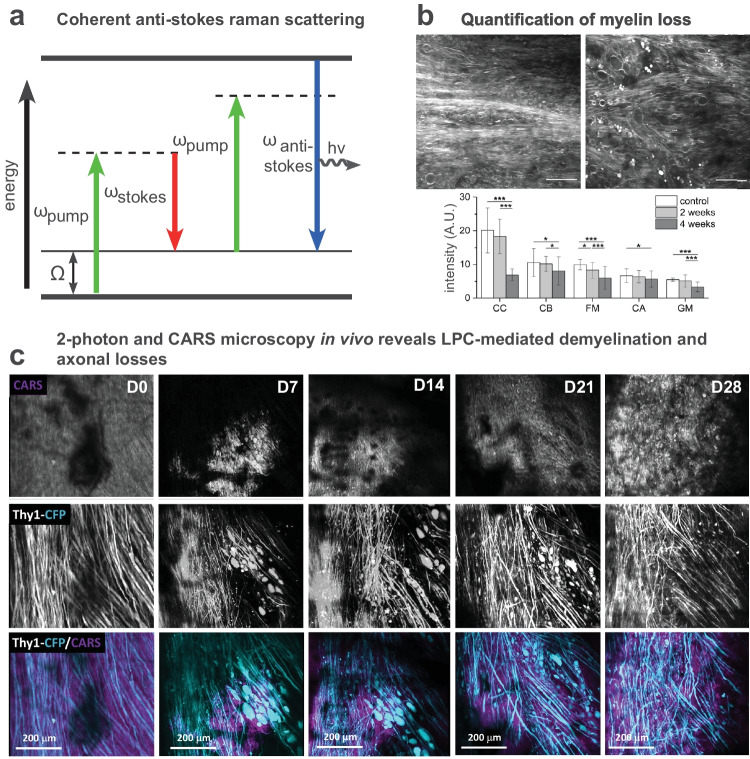
CARS microscopy to study lipid-rich myelin in vivo. **a** Principle of coherent Anti-Stoke Raman Scattering (CARS) microscopy. **b** A reduction of the CARS signal indicates loss of myelin after repeated imaging sessions (modified from [75]). **c** Longitudinal imaging reveals LPC-mediated myelin and axon degeneration and rescue (modified from [19])

**Fig. 3:**
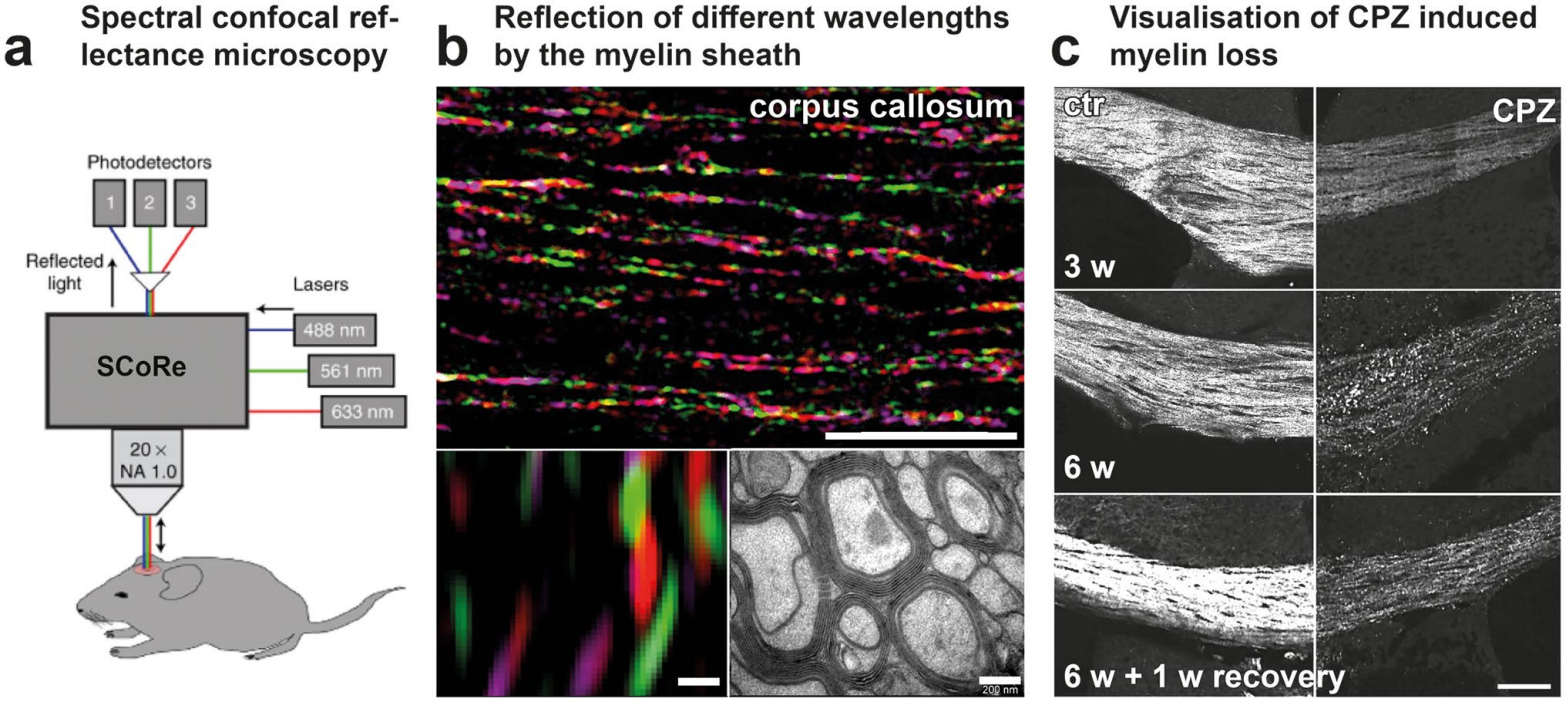
Label-free in vivo imaging of myelinated axons by spectral confocal reflectance (SCoRe) microscopy. **a** Principle of SCoRe microscopy (modified from [72]). **b** Imaging of compact myelin structures using SCoRe microscopy. **c** SCoRe microscopy of myelin and its loss after CPZ-induced demyelination over 6 weeks, plus one week recovery. **b** and **c** (modified from [73])

MS is a complex pathology, involving many cell types and mechanisms that differ according to disease phases and affected CNS regions. The optical properties of conventional light microscopy did not enable assessing cellular responses in deep tissues of living animals. The development and implementation of non-linear fluorescence microscopy in particular two-photon laser-scanning microscopy (2P-LSM) have thus revolutionized the characterization of cellular events in vivo (Fig. 1a). In addition, combined with surgical implantation of cranial or dorsal windows for longitudinal chronic imaging, transgenic mice expressing fluorescent proteins of various spectral properties in specific cells populations appeared very valuable to track the dynamics and interactions of cells of interest (Fig. 1b). The kinetics of cell activation, their infiltration through the blood vessel walls, and the Ca2^+^ dependence of immune-related physiological events have been demonstrated. Moreover, the implementation of free-labeling in vivo microscopies such as CARS (Coherent Anti-Stokes Raman Scattering) (Fig. 2a) or SCoRE (Spectral Confocal REflectance microscopy) (Fig. 3a) allowed to visualize changes of the myelin sheath during demyelination and remyelination processes.

#### Cellular Mechanisms, Interaction and Dynamic of Cells

MS is driven by autoreactive T cells originating either from memory T cells reactivated by contacts with antigens in the environment or from naïve T cells educated at the cervical lymph nodes against myelin or neuronal antigens by Antigen Presenting Cells (APCs) such as Dendritic Cells (DCs). After priming, these CNS-specific reactive CD4^+^ and CD8^+^ T cells cross the Blood-Brain Barrier (BBB) and are reactivated by APCs inside the parenchyma [46]. Their migration across the BBB is a crucial and precise step. Real time two-photon (2P) imaging of transferred encephalitogenic CD4^+^ T cells transduced with green fluorescent protein (GFP) established that these cells arrest preferably at leptomeningeal microvessels before crawling, specifically along VE-cadherin adherens junctions of endothelial cells and, cross the BBB by diapedesis [47, 48]. In EAE, T cells are involved before the onset of clinical deficits and their activity is shown as Ca^2+^-dependent. Crossing the BBB implies an increase of their Ca^2+^ signal that correlates with translocation of Nuclear Factor of Activated T cells (NFAT) into their nuclei. Moreover, they sustain elevated Ca^2+^ signals while searching for APC contacts, which emphasizes the key role of Ca^2+^ in their chronic reactivation and proliferation [49, 50]. Different subsets of myelin-reactive CD4^+^ are involved in EAE pathogenesis such as T helper 17 (Th17) and 1 (Th1), especially at the onset and chronic phases. 2P-imaging revealed that CD11c^+^ APC cells accumulate in the perivascular space at the onset and peak of EAE, where they establish long-lasting contacts preferentially with Th17 cells to promote their tissue invasion [51]. Unlike Th1 cells, Th17 cells adopt different motility pattern with a reduced mean velocity at the peak of EAE symptoms as well as in the chronic phase. Slow kinetics correlated with a higher interaction rate with neuronal processes in demyelinated areas. Direct contacts of Th17 cells with unmyelinated axons or neuronal somata lead to a Ca^2+^ increase, preceding axonal degeneration [52]. Beside neurons, Th17 cells can also make direct contacts with OLs hence causing cell death and demyelination through an integrin CD29 dependent mechanism involving glutamate release and change in lipid biosynthesis [53]. Interaction of cytotoxic CD8^+^ T cells with myelin-peptides on OL is instead not sufficient to induce Ca^2+^ rise in neighboring axons nor to exacerbate axonal injuries [54].

In addition to their direct interaction with parenchymal cells, Th17 cells also release IL-17 cytokine which induces the recruitment of myeloid cells, such as neutrophils or monocytes, thought to worsen inflammation [55, 56]. Inducing EAE in CD11c-EYFP//LysM-EGFP mice allowed the identification of cell subsets as well as their time scale accumulation in SC lesions (Fig. 1b, c). EGFP^+^ neutrophils and monocytes are first recruited inside the parenchyma where they progressively differentiate into monocyte-derived CD11c^+^ cells concomitantly with the stabilization of axonal degeneration and clinical signs [57, 58].

As the CNS resident innate immune cells, the involvement of microglia cells plays an important role in the evolution of MS pathophysiology. The recruitment of CX_3_CR_1_-EGFP^+^ microglia cells monitored by in vivo 2P-LSM microscopy showed their rapid involvement with morphological changes in presence of myelin debris and degenerated axons [59]. Moreover, the number of CD11c^+^ microglia, a specific subset of activated microglia, increases drastically during EAE. Even if these cells share common features with monocyte-derived CD11c^+^ cells such as the expression of the MHC-II complex, they are not considered APCs for T cells reactivation [60]. CD11c^+^ microglia are recruited at the plaques where they adopt a phagocytic phenotype and digest axonal debris during the acute phase of EAE [57]. Heterogeneity of microglia phenotypes is reflected by their CNS region-specific response to demyelination, especially in the forebrain. 3D analysis of the morphologies of single microglial cells revealed that microglia in the hippocampus are the first to show hypertrophic cell bodies at the early stages of the disease; in the cortical layers instead, phagocytic activity is mainly observed in layer V microglia along with reduced lengths of their processes and decreased ramification. Layer 2/3 microglia are instead only weakly activated upon demyelination but adopt a hyper-ramified morphology during remyelination [61]. Interestingly, beside microglia, mononuclear phagocytes — including monocyte-derived cells, dendritic cells — exhibit different inflammatory phenotypes along with disease progression. By translating polarization of phagocytes into distinct fluorescent signal, it appears that these cells acquired a pro-inflammatory phenotype at the onset phase with iNOS release prior to switch to an anti-inflammatory one defined by arginase secretion during the remission phase [62]. In addition, microglia cells preferentially contact myelinated axons at the nodes of Ranvier and modulate by K^+^ release. The altered K^+^ in demyelinating plaques promotes IGF1^+^ pro-regenerative microglia cells, which increase their specific contacts with axons in order to remyelinate and to repair the lesions [63]. Similarly, time-defined depletion of the subset of anti-inflammatory regulatory CD4^+^ T cells (Treg cells) during the chronic phase of EAE exacerbates EAE clinical outcomes by increasing in vivo pro-inflammatory-cytokines secretion, proliferation, and motility of T cells. 2P-imaging of the distribution and motility patterns of Treg cells revealed that they might mediate recovery via suppression of Ca^2+^ signaling in Th17 cells, which reduced their contacts with APCs’ [64, 65]. In all cases, inflammatory conditions appear inhospitable to generate new OLs. Remyelination indeed fails in demyelinated plaques due to inefficient recruitment of OPCs, whose proliferation, differentiation, and subsequent maturation into myelinating OLs are impeded [66, 67]. It is therefore important to elucidate, beyond the proliferation itself, how these OPCs could be efficiently driven to differentiate into myelinating OLs.

#### Free Labeling Microscopy to Visualize the Myelin Sheath

Monitoring the fate of myelinating OLs and subsequent deposition of myelin is a key challenge of MS research. Treatment of cognitive defects and functional disabilities consecutive to the pathological myelin loss relies on the precise understanding of the cellular events and interactions regulating demyelination and remyelination. The recent development of several high-resolution and label-free in vivo imaging paved the way for dynamically characterizing myelin structures, both in animal and human tissues. While optical coherence microscopy (OCM) [68] and third-harmonic generation (THG) [69, 70] are demanding optical methods, CARS (Fig. 2a) and SCoRE (Fig. 3a) microscopies can readily be implemented into an experimental routine. CARS is based on the specific symmetrical vibrational signature of CH2 bonds contained in lipids of myelin sheath [71]. Local areas of decreased CARS signal have been reported from axons in the CC and spinal cord (SC) of EAE immunized mice (Fig. 2b) reflecting demyelination and myelin loss [59]. Similar observations are obtained with SCoRE microscopy, based on the specific wavelength dependent reflection of compact myelin (Fig. 3a, b). 2P imaging in combination to SCoRE microscopy on Thy1-EYFP mice showed a drop of SCoRE signal from unmyelinated Thy1^+^ axons [72]. In the CC of CPZ-treated mice, SCoRE imaging revealed a reduction of compact myelin in rostral and caudal areas, in parallel to an accumulation of myelin debris (Fig. 3c). 3D reconstruction of the acquired images depicted spatial colocalization with phagocytic cells cleaning the environment of myelin debris [73].

The mechanisms whereby demyelination is initiated are still unclear. During the relapsing phase of EAE and progressive demyelination, the retraction of paranodal myelin along lesion borders is observed as an early event, hence exposing and/or disorganizing neuronal voltage-gated Kv1.2 channels and subsequently altering K^+^ release inside the lesions [63, 74]. In support, the deposition of droplets-like myelin debris along myelinated axons seen by CARS microscopy might be considered one of the most initial stages of demyelination before the loss of OLs themselves, given that the magnitude of droplet accumulation being inversely correlated to myelin sheath thickness [75]. Once myelin damage is initiated, in vivo monitoring of the first events of demyelination by CARS established that myelin sheath disruption is sequential and follows a precise pattern of degeneration, organized into well-defined steps and preceding the axonal loss. From wavy shape to bubble-like swelling, myelin sheath rapidly forms disconnected vesicles containing axonal pieces, until myelin debris accumulate in the lesion site [19]. Longitudinal multimodal 2P+CARS microscopy next confirmed that demyelination can trigger axonal degeneration, while axonal regeneration precedes remyelination in LPC-induced focal demyelination (Fig. 2c). Ca2^+^ dependent mechanisms involving cPLA_2_ and calpain enzymatic activities have been proposed to support these demyelinating and neurodegenerative processes [76]. Spectral differences in CARS signals were reported also in post-mortem tissues of patients, in particular in regions named as “normal-appearing white matter” (NAWM) surrounding demyelinating MS plaques that were initially thought unaffected by the disease at this stage of progression [77]. In the EAE mouse model, NAWM regions exhibit molecular disorganization of myelin lipids as well as discrete changes in biochemical signatures despite the lack of obvious alterations of the myelin sheath inside these regions [78].

### Recent Advances of Non-Linear Optical Microscopy to Improve Single-Cell Resolution for Preclinical Imaging

Nowadays, IVM of animal models has become pivotal to investigate and understand the molecular and cellular processes of MS, but its improvement remains a challenge. 2P-LSM gave access to deep CNS tissue. However, optical tissue properties such as auto-fluorescence or photon scattering limit imaging depth and image quality. Compared to 2P-LSM which uses 800–1100 nm excitation wavelength, three-photon (3P) microscopy requires longer excitation wavelength near 1300 nm or 1700 nm, also referred to as “Short Wavelength Infrared Range” (SWIR). Thereby, 3P microscopy provides an improved signal in deep tissue by lowering focal plane background and increasing the signal-to-noise ratio [79]. 3P excitation at 1300 nm allowed recording of neuronal Ca^2+^ signals on intact brains directly through the skull with a depth range of 500 µm into the cortex and even into the hippocampus of awake mice [80–82]. 3P microscopy has to be used cautiously due to increased water absorption within the SWIR window. Whereas higher laser intensities are required at the focus to achieve significant fluorophore excitation, researchers have to deal with increased risk of tissue heating as well as enhanced photobleaching [83]. Yet laser excitation conditions at 1340 nm have been successfully established to perform functional multicolor imaging over a depth of 1200 µm in the adult mouse brain (Fig. 1d) while limiting photobleaching [84]. Because THG signal can easily be generated in the visible spectrum from the wavelength used for 3P excitation, the combination of 3P microscopy with THG appears as a promising non-invasive tool for the multiparametric characterization of the fluorescently labeled cellular microenvironment simultaneously to the label-free imaging of the myelin coverage inside thick and diffusive tissue [85] (Fig. 1d).

Furthermore, the adaptive optics (AO) technology, used by astrophysicists to overcome the fluctuations of atmospheric conditions during star gazing is another way to compensate scattering and optical aberrations introduced by refractive index mismatches inside biological tissues. Dynamic modulation of the light phase can correct biological distortions of the wavefront to recover an optimal image resolution [86]. AO has been used to record neuronal activity at bouton level deeply in the cortex of awake mice. It was also used to resolve fine structures in retinal layers, to perform functional Ca^2+^ imaging of neuronal soma and dendrites as well as to study the structural alterations of microglia processes in a pathological context [87, 88]. When applied on 3P recordings, AO corrections enable visualization of cortical spines and dendrites with a depth of 1.4 mm [89]. Finally, all IVM images can be post-treated with Artificial Intelligence (AI)-based computational methods to improve their quality. Deep learning algorithms can be used to denoise fluorescent images or to recover high resolution 3D images from blurred and light-scattered images of deep tissues [90, 91]. AI might also compensate for the limited availability of fluorescent labels to selectively identify cells or structures of interest in vivo. The machine can be trained to associate the endogenous contrast signatures with the corresponding fluorescent label from the same objects identified in sets of paired images acquired by transmitted light microscopy (e.g. bright field, phase or differential interference contrast) and by fluorescence microscopy. The in silico labeling can then be performed a posteriori to identify cell types from unlabelled IVM images [92, 93]. However, this approach has so far not been tested in vivo.

### Animal Models and Whole-Body Preclinical Imaging: Development and Improvement of Diagnostic Tools

**Fig. 4:**
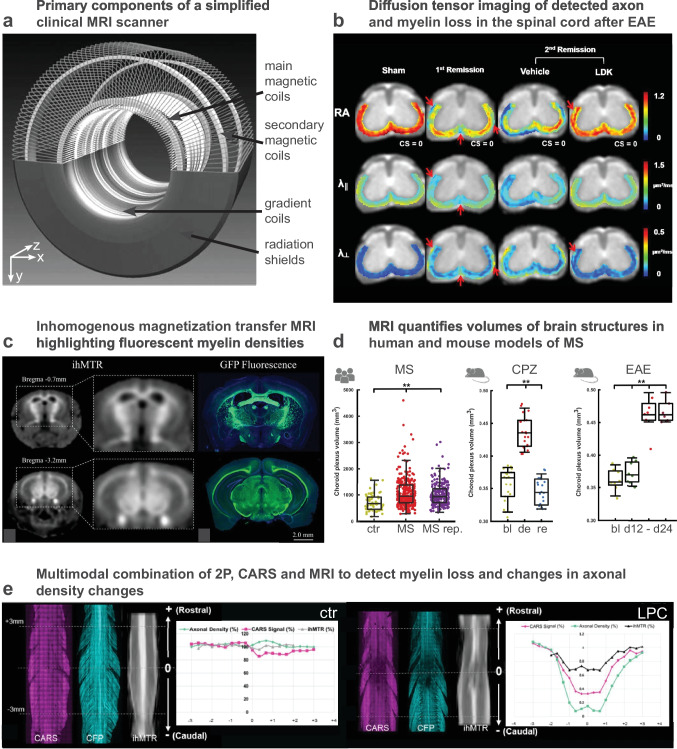
Magnetic resonance imaging of myelin and its combination with light microscopy. **a** Primary components of a simplified clinical magnetic resonance imaging scanner (modified from [154]). **b** Magnetic resonance-based diffusion tensor imaging of the mouse spinal cord after EAE (modified from [155]). **c** Inhomogeneous magnetic transfer (ihMTR) imaging to visualize white matter in mice as compared to myelin density evaluated from GFP reporter expression in a transgenic animal [156]). **d** MRI-based quantification of the choroid plexus volume as an index of MS disease progression in human and mouse models of MS (modified from [157]). **e** Combined evaluation of axonal myelin and LPC-evoked myelin loss in the mouse spinal cord by MRI (ihMTR), CARS and 2P microscopy (modified from [158])

**Fig. 5:**
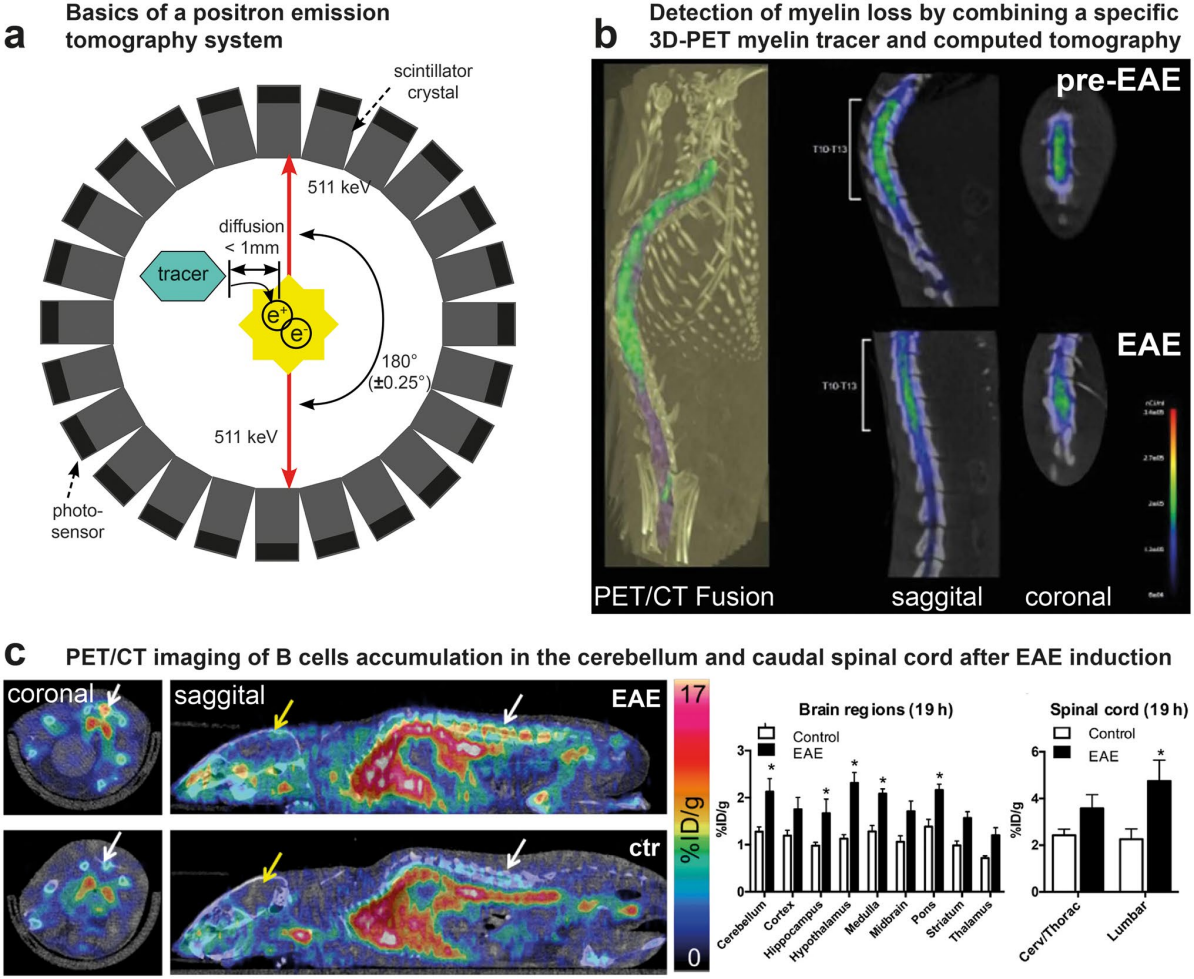
Positron emission and computer tomography (PET and CT) to follow myelin loss in animal models of MS. **a** Physics of a PET system (modified from [159]). **b** Combined 3D-PET/CT scan of a rat spinal cord before and after induction of EAE. The reduction of the [11C]MeDAS signal indicates myelin loss. **c** EAE-evoked inflammation can be followed in mice by PET using a [64Cu]-rituximab tracer that specifically binds to B cells and highlight the preferential and localized accumulation of B cells in the cerebellum and lumbar spinal cord (modified from [131])

Although IVM enabled great advances for neuroimmunology, the constraints associated with invasiveness, limited imaging depth or restricted fields of view have limited the study of the systemic components of the disease as well as the dynamics of its spread in the whole CNS. It is, however, clear from both experimental imaging of animals and biomedical imaging of patients that the initiation site of MS pathology is not completely random. It is rather following an orchestrated sequence of progression from the deepest white matter regions to the most superficial grey matter ones [94], ultimately leading to patterned brain structure atrophy [95]. Magnetic resonance imaging (MRI) (Fig. 4a), positron emission tomography (PET) (Fig. 5a), or computed tomography (CT) are clinical imaging modalities that have nurtured these observations in human patients. Although their extensive use in preclinical research has for long been challenged by their intrinsically limited spatial resolution (typically mm^3^) [96], recent technological breakthroughs in the field of magnets and antennas [97, 98] resolving scintillator detector or selective high-contrast agents [99] have brought these modalities on the front stage to provide complementary information to IVM on rodent models.

In MRI, the magnetization transfer ratio (MTR) and the bound proton fraction f* (also referred to as bound pool fraction f and macromolecular proton fraction (MPR)) are reliable readouts for quantitative mapping of myelin content and have been successfully employed to quantify myelin loss in EAE experimental animals [100]. MTR and f* show a local decrease within the lesions and are modulated according to the demyelination severity or the loss of the cellular matrix [101]. Moreover, the analysis of water diffusion maps provides information on the microstructure of the WM (Fig. 4b) and is correlated with post mortem immunodetection of MBP, both in the SC and in several other brain regions such as CC, cortex, and hippocampus [102, 103]. Demyelinated plaques can furthermore be classified into several subtypes whose contrasts appear different in T2-weighted images. Changes in magnetic susceptibility globally reflect immune cell infiltration with a particular bias toward the accumulation of iron-rich macrophages inside the lesions [104, 105]. Cellular specificity of these endogenous contrasts, however, is still poor and requires more investigations [106, 107].

Several exogenous labeling strategies have been evaluated to better characterize cell infiltration dynamics, hence the time course of MS pathology. Because macrophages are endowed with phagocyting capacities, their in vivo labeling can be achieved by systemic injection of ultra-small particles of iron oxide (USPIO) 24 h prior to MRI imaging session. Many iron-rich areas can thus be highlighted in macrophage-enriched demyelinated lesions named contrast enhancing lesion (CEL). Their number is specifically high between the onset and the peak of EAE disease as well as during the progressive phase of the TMEV mouse model [108, 109].

Similarly, attempt to localize the earliest events of leukocytes infiltration in the CNS was achieved by targeting microparticle iron oxide-labeled P-selectin antibodies (MPIOs-αPselectin) to endothelial cells since their upregulation of P selectin expression is thought to affect the invasion of myelin-specific T cells through the BBB. Intravenous injection, a few minutes before MRI imaging, thus provides strong evidence of early and localized vascular inflammation, biased toward the ventral regions of the SC and announcing initial and secondary relapses [110].

Because T cells are considered disease initiating cells, efforts have been made to label them ex vivo prior to their transfer in recipient animals. In rats, at the first sign of hindlimb paralysis following injection of myelin reactive T cells, MRI imaging outlined their selective accumulation in the sacral SC. If rats had already been primed with the disease, the cells then also spread inside the brain [108]. Given its detection sensitivity, MRI requires either accumulation of labeled cells at high densities or the loading of cells with high concentration of labeling agents. Whereas the first requirement is met in the case of phagocytic cells and entirely relies on biological processes, the second requirement can instead be met experimentally, by adjusting ex vivo conditions until the proper SPIO intracellular concentration is achieved. Noteworthy, careful assessment of cell survival and cell functions have to be made depending on the pathophysiological mechanism under evaluation.

When cell tracking is concerned, lessons from the field of cancer research have pointed out that positron emission tomography (PET) scanning has to be preferred over MRI for high sensitivity detection of tracing amount of labeling agent [111]. Initially developed to monitor accumulation of fluordesoxyglucose (FDG) in glycolytic tumors, PET scanning first appeared as a useful technique to monitor inflammation driven hyperglycolysis in the CNS of rodent models of MS [112, 113]. Yet the development of 1,4-bis(p-aminostyryl)-2-methoxy benzene (BMB), a BBB permeant organic molecule selectively binding to myelin, opened the way to the orginal development of (11)C-radiolabeled BMB to be used in vivo to image CNS myelin by PET in baboon [114].

Given the extreme sensitivity of the technique to evaluate picomolar concentrations of specific tracers, new generations of myelin-specific contrast agents were developed to serve MS research. [^11^C]MeDAS and [^11^C]-PIB were thus proven to reliably report myelin status during demyelination and remyelination in both LPC and EAE (Fig. 5b) rodent models [115, 116], but also in non-human primates (NHP) to accelerate the transfer to human clinics [117]. Although physical laws intrinsically limit the spatial resolution of PET to millimeter scale, medium-sized and large animal models (from rats to monkeys) can fully take advantage of the high specificity of the tracers, along with the absence of radioactive background, to quantitatively characterize the fate of myelin sheath in different brain structures [117] and at different stages of the disease [116].

Given the prevalent role of inflammation in disease evolution and its sparse localisation evidenced by MRI, selective PET markers for inflammatory cells have been developed to characterize the cell populations involved and their dynamics during the course of the disease. Binding of folate as [^18^F]-folate to the folate receptor β (FR-β, FOLR2) was thus used to label activated macrophages [118], while the purinergic receptor P2X7R was targeted by [^11^C]-labeled antibodies [119] or [^11^C]-SMW139 ligand [120] to monitor accumulation of the deleterious pro-inflammatory microglia in the brain and the SC. Other promising tracers for microglia consisted in [^18^F]-VC701 or [^18^F]-DPA-714, two different ligands of the 18kD-translocator protein (TSPO) whose expression is increased in glial cells upon parenchymal infiltration of peripheral inflammatory cells. Although [^18^F]-VC701 and [^18^F]-DPA-714 indeed accumulated into the demyelinated lesions previously determined by T2*-weighted MRI into the cortex, hippocampus, cerebellum or cervical SC [121, 122], the cellular specificity of the contrast remains uncertain based on recent reports outlining the overexpression of TSPO, not only in microglia but also in astrocytes, in vascular endothelial cells and activated neurons [123].

Because radioactive tracing can be implemented without significantly affecting the 3D structure of the molecule of interest, PET scan imaging can also be used to evaluate in situ the distribution and function of enzyme or bioactive metabolites controlling neuroinflammation [124]. Radiolabeled matrix metalloproteinase 9 (MMP-9) inhibitors were thus used to visualize enzymatic activity in the brain and SC of EAE mice. MMP9 PET-imaging demonstrated the earlier detection of lesions and leukocytic infiltration than what achieved with standard gadolinium contrast enhanced MRI scans [125]. Because sphingosine 1-phosphate receptors (S1PR) present on lymphocytes regulate their egress from lymphoid tissues into circulation and into CNS [126, 127] and because microglial S1PR also regulate activation state and neurotrophic potential of microglial cells [128], this receptor was also targeted with the hope to visualize its distribution in the earliest steps of neuroinflammation. Several generations of S1PR ligands were thus developed to optimize their brain up-take [129] hence offering new tools to dynamically monitor the reported agonist effect of fingolimod, the first approved oral therapeutic drug for treating relapsing MS [126].

Because both T cells [130] and B cells [131, 132] specific radioligands have been developed for PET imaging (Fig. 5c), it should be possible in the future, at least in animal models of MS, to compare the contrast distribution in images stacks successively obtained with S1PR ligands with the one observed with cell type-specific ligands in order to clarify the preferential localization of the S1PR receptor on microglia or lymphocytes. Such multi-tracer approaches have been successfully implemented to explore the diversity and spatial heterogeneity of multiple pathomechanisms in rodent models of other neurodegenerative diseases [133, 134]. Like for IVM, AI algorithms can also be used in PET imaging to improve the quantitative measurement of each radiotracer concentration depending on its localization in the body [135]. Therefore, it can be envisioned that the relative densities of the most relevant cells for neuroinflammatory lesions will soon be dynamically examined by PET during the course of the pathology and at every location of the CNS instead of being limited to the superficial SC layers with IVM. The observed modulation of neuroimmune interactions by disease modifying treatments will then help to unravel their cellular targets and mechanisms of action in view of dosage optimization and therapeutic window determinations prior to clinical use.

In this prospect, NHP models of MS [136] will be of the highest value not only to counterbalance the modest spatial resolution of the PET imaging technique but also to improve the clinical relevance of the model with regard to the human pathophysiology and its response to therapeutic intervention. Improvements of PET scan detection technologies today allow to establish imaging protocols requiring reduced exposure to ionizing radiation, and therefore are compatible with longitudinal studies over several weeks on weekly basis [133, 137–140].

**Fig. 6:**
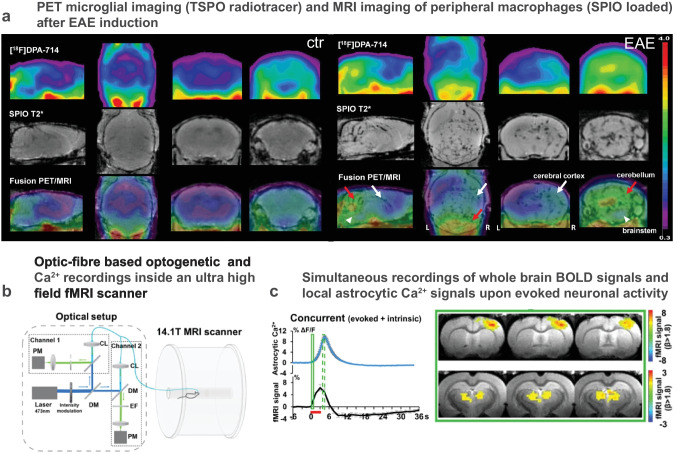
Multimodal imaging of animal models bridges the gap to the clinic. **a** Multimodal PET-MRI successfully outlines areas containing macrophages loaded by intraveinously injected superparamagnetic iron oxide (SPIO) and seen by MRI as well as activated microglia/astrocytes labeled by [18F]-DPA-714 binding to 18 kD-translocator protein (TSPO) detected by PET (adapted from [141]). **b** Simultaneous fiber-based fluorescence photometry and MRI to record genetically encoded physiological probes (such as glial Ca2^+^ signals using viral infections with GCaMPs) together with neuronal activity as determined by the blood-oxygen level dependent (BOLD) MRI signal. **c** The analysis of correlation between astrocytic Ca2^+^ signals and BOLD outlines thalamocortical activity patterns. **b** and **c** (modified from [147]

Due to the complex nature of the technique and the high costs involved, PET imaging will probably never replace MR imaging to study MS, but it can excellently complement MR imaging by bringing molecular specificity to in vivo evaluation of CNS inflammatory plaques, in particular when therapeutic drug development is concerned. The best option to really characterize inflammatory cell recruitment with respect to demyelination at the scale of the entire brain or SC is certainly to perform multimodal PET-MRI (Fig. 6a).

### Multimodality for Complete Investigation of NHP Models and Clinical Transfer

Besides giving access to detailed anatomical structures and to the myelination status through endogenous MRI contrast, the use of SPIO allowed quantifying the differential recruitment of peripheral macrophages in the hippocampus, thalamus, and cerebellum by MRI, while the use of [^18^F]DPA-714 PET gave access to the corresponding activation status of resident glial cells in the same regions by PET [141]. These first experiments clearly demonstrated the feasibility and the interest of multimodal approaches for repeated imaging sessions with a time-span of 24 h (Fig. 6a). Given the tremendous technological efforts made for the development of multimodal scanner [142] and multimodal probes [143] to simultaneously acquire PET and MRI signals, a middle term blue-sky project will most likely consist in evaluating in real time the dynamic interactions between CNS resident microglia and peripheral macrophages, or between microglia and autoreactive B or T cells in NHP models of MS [136]. On top of providing fundamental knowledge on the neuroinflammatory cascades regulating human health, this same technology will undoubtedly prove useful to optimize pharmacological treatments delivery [144] or to track the distribution of myelin specific chimeric antigen receptor T cells (CAR-T) either injected systemically [145] or generated in vivo through viral therapy [146]. Noteworthy, the blood-oxygen level-dependent (BOLD) signal accessible from functional MRI scans can eventually be used to assess the impact of neighboring immune cell densities on the local neuronal activities, either resting state or evoked ones. Such study would thus provide insights into the origin of the neurological deficits.

In this prospect, recent methodological studies indicated that the cellular origin of the recorded signals can be detected in the same animals using IVM along with viral transgenic expression of fluorescent genetically encoded Ca^2+^ indicators and/or intraveinous injection of fluorescent contrast agents [147, 148]. In this case, multimodality can be achieved after precise implantation of cranial glass windows to standardize animal repositioning across successive imaging modalities [148]. The integration of such multiscale data across spatial resolution can benefit from in silico cross-modality imaging (CMI) pipelines to accurately co-register the different images datasets prior to semi-automated quantification [149]. Alternatively concomitant imaging of the same biological events was successfully achieved by the use of an MRI compatible optic fiber-based photometry device [147] (Fig. 6b). Because the development of fiber-based 2P and CARS endoscopes is currently a field of intensive research [150–152], it is tempting to speculate that fluorescence IVM with subcellular resolution will soon be possible inside PET/MRI scanner to benefit from the advantage of each imaging modality on the same animal over recurrent imaging sessions during the course of a disease and its treatment.

## Conclusion

While optical imaging has always been used in biological research to collect information on small transparent organisms, co-development of non-linear fluorescence imaging and genetically encoded probes over the past 25 years has allowed to dissect the dynamical interactions of identified individual cells in real time in situ in the superficial parts of small and large mammals. These technologies were particularly instrumental to image cell populations identified as the most important ones for neuroinflammatory lesions initiation and evolution, in particular to describe their sequential recruitment. Because these cells originate from extra CNS location and spread all over the body, their site of infiltration and the distribution of lesions remains poorly predictable. In order to build a global view of disease progression, whole body imaging data are needed. Tremendous progressions of the use of clinical MRI and PET scanner in all fields of medicine have nurtured important breakthroughs in the field of targeted contrast agents for both modalities. Because small animal models were required to evaluate the quality, selectivity, and toxicity of these agents, high resolution preclinical versions of the clinical instruments have been developed, pushing the technologies to their best performances. As intrinsic physical limitations still restrict the spatial resolution to the millimeter range with rodent models of MS, the use of NHP models in the neuroimaging research is highly relevant based on both, their brain size and their phylogenetic similarity with humans allowing better clinical translation of experimental observations even if their use is limited by ethical and cost reasons. Multiparametric information can indeed be collected from the same animals at different time points which strengthens the statistical significance of all acquired results. In the context of MS research, similarly to what happens in the field of cancer, the future of intravital neuroimaging will most likely include advanced multimodal equipment. With MRI, resolutive anatomy, myelin assessment, and functional imaging will be possible throughout the body and the whole CNS in order to localize active lymphatic nodes and pathological lesion sites. These will then be characterized at the cellular and molecular level thanks to selective PET tracers with high sensitivity and specificity on a millimeter scale and an hour time resolution. IVM will instead provide submicron and sub-second time resolutions in submillimetric fields of view while offering the advantage of using genetic tools to dissect the cell/organelle specific origin of the recorded signals. If implemented in a label-free Raman spectroscopic mode [150, 152, 153], thin endoscopes implanted in the vicinity of MRI detected lesion will offer the unique advantage to detect the earliest structural changes of myelin sheath in response to immune cell interactions or in response to innovative remyelinating treatments. Although these terabytes of images could easily overwhelm the brain of human analysts, they can nowadays be used to train and to feed AI algorithms to help predicting disease evolution for the sake of therapeutic intervention and the benefits of patients.

## Supplementary Information

Below is the link to the electronic supplementary material.

Supplementary file1 (PDF 517 kb)

Supplementary file2 (PDF 517 kb)

Supplementary file3 (PDF 525 kb)

Supplementary file4 (PDF 508 kb)

Supplementary file5 (PDF 517 kb)
